# Functional Connectivity in the Left Dorsal Stream Facilitates Simultaneous Language Translation: An EEG Study

**DOI:** 10.3389/fnhum.2016.00060

**Published:** 2016-02-19

**Authors:** Stefan Elmer, Jürg Kühnis

**Affiliations:** Auditory Research Group Zurich (ARGZ), Division Neuropsychology, Institute of Psychology, University of ZurichZurich, Switzerland

**Keywords:** simultaneous interpreters, EEG, functional connectivity, dorsal stream, lexical decision task

## Abstract

Cortical speech processing is dependent on the mutual interdependence of two distinctive processing streams supporting sound-to-meaning (i.e., ventral stream) and sound-to-articulation (i.e., dorsal stream) mapping. Here, we compared the strengths of intracranial functional connectivity between two main hubs of the dorsal stream, namely the left auditory-related cortex (ARC) and Broca’s region, in a sample of simultaneous interpreters (SIs) and multilingual control subjects while the participants performed a mixed and unmixed auditory semantic decision task. Under normal listening conditions such kind of tasks are known to initiate a spread of activation along the ventral stream. However, due to extensive and specific training, here we predicted that SIs will more strongly recruit the dorsal pathway in order to pre-activate the speech codes of the corresponding translation. In line with this reasoning, EEG results demonstrate increased left-hemispheric theta phase synchronization in SLI compared to multilingual control participants during early task-related processing stages. In addition, within the SI group functional connectivity strength in the left dorsal pathway was positively related to the cumulative number of training hours across lifespan, and inversely correlated with the age of training commencement. Hence, we propose that the alignment of neuronal oscillations between brain regions involved in “hearing” and “speaking” results from an intertwining of training, sensitive period, and predisposition.

## Introduction

Different experimental approaches have demonstrated that the speech perception and production systems are tightly coupled (Liberman and Mattingly, 1985). Such an interrelationship can, for example, be observed in congenitally deaf individuals who often achieve only rudimentary speech competence (Smith, 1975). On the other side, altered auditory feedback has a disruptive effect on speech production (Yates, 1963; Houde and Jordan, 1998), and subjects suffering from dysarthria or Broca aphasia are often accompanied by language comprehension deficits (Hustad and Beukelman, 2002; Davis et al., 2008).

The arcuate fasciculus (AF) is part of the dorsal processing stream (Makris et al., 2005; Hickok and Poeppel, 2007) and connects the posterior bank of the supratemporal plane with the ventral prefrontal cortex (i.e., Broca’s area; Makris et al., 2005; Rilling et al., 2008). The AF is strongly lateralized to the left hemisphere (Catani and Mesulam, 2008; Glasser and Rilling, 2008) and integrates auditory information with the frontal articulatory system. Otherwise, the inferior longitudinal fasciculus (ILF) is part of the ventral stream (Hickok and Poeppel, 2007), stretches from occipitotemporal regions toward the temporal pole (Dick et al., 2014), and constitutes the anatomical correlate underlying sound-to-meaning mapping (Almairac et al., 2014).

The medial-dorsal bank of the supratemporal plane is part of the auditory-related cortex (ARC; Geschwin and Levitsky, 1968; Galaburda et al., 1978; Steinmetz et al., 1991; Jäncke and Steinmetz, 2004) and behaves as a computational hub by segregating spectrotemporal information (Griffiths and Warren, 2002). This brain region is essentially involved in phonetic processes (Jäncke et al., 2002; Osnes et al., 2011; Elmer et al., 2012), language comprehension functions (Tzourio et al., 1998a,b) and can serve as a marker for left hemispheric language specialization (Tzourio et al., 1998a,b; Josse et al., 2003; Josse and Tzourio-Mazoyer, 2004). In addition, due to its anatomical location, the ARC acts as an interface between sound-to-meaning and sound-to-articulation mapping mechanisms. Broca’s region is engaged in a variety of different cognitive- (Fedorenko et al., 2012; Makuuchi and Friederici, 2013), linguistic- (Grodzinsky and Friederici, 2006; Grodzinsky and Santi, 2008), and speech-related functions (Eickhoff et al., 2009; Moser et al., 2009). There is even evidence indicating that this brain region, in association with other circuits, is implicated in the orchestration of the articulatory system and of paramount importance for the planning and execution of articulatory processes (Eickhoff et al., 2009).

SIs are specifically trained in translating a source- into a target language quasi simultaneously (Elmer, 2012). During such a task, auditory speech cues have to reliably be associated with lexical/semantic representations along the ventral stream as well as to be coupled with the dorsal articulatory system with minimal time delay. Since the ARC has previously been proposed to behave as an interface between sound-to-meaning and sound-to-articulation mapping mechanisms (Hickok and Poeppel, 2007), and Broca’s region constitutes a main hub within the articulatory system, here we evaluated functional connectivity between these two brain regions in professional SIs and multilingual control subjects while the participants performed a mixed and unmixed semantic decision task.

## Materials and Methods

### Participants

We measured a sample of professional SIs [principally interpreting from English (L2) to German (L1), totally 12, 10 women, mean age = 37.9 years, SD = 6.9 years] and multilingual controls participants (totally 12, 9 women, mean age = 39 years, SD = 4.7 years). All participants were consistently right-handed, as revealed by the Annett Handedness Inventory (Annett, 1970), and were native German speakers with a good English listening comprehension (DIALANG^1^). The two groups were matched for age, gender, L2 proficiency, age of L2 acquisition, and education (i.e., University degree). None of the participants reported a history of neurological, psychiatric or audiological disorders (Home Audiometer Software^2^) and all participants denied consumption of illegal drugs or regular medication. The participants were paid for participation, the local ethics committee approved the study, and written informed consent was obtained. Table 1 provides an overview of the biographical and behavioral data.

**Table 1:** **Autobiographical and behavioral data of the participants**.

Subjects	Gender	Age	AOA [E]	Years of experience as SI	Listening [E]	AOA [foreign languages]
1	f	39	13	10	c1	French (5, 4), Hebrew (17, 4), Italian (17, 2), Spanish (34, 3)
2	f	46	12	24	c1	French (5, 3), Spanish (23, 3)
3	f	40	16	8	c1	French (4, 3), Italian (30, 3)
4	f	44	14	20	c1	French (12, 4), Spanish (16, 3), Italian (25, 3)
5	f	42	10	15	c1	French (12, 4), Italian (22, 4), Spanish (32, 3)
6	f	34	11	4	c1	French (20, 4), Italian (22, 2)
7	m	40	14	9	c1	French (12, 4), Spanish (21, 3)
8	f	48	14	8	c1	French (birth, 4), Spanish (21, 4), Italian (23, 3), Portuguese (47, 2)
9	f	35	12	8	c2	Italian (15, 3), French (13, 3)
10	f	26	6	1	c2	French (10, 3), Spanish (22, 3), Serbian (26, 2)
11	m	33	14	3	c2	French (14, 3), Italian (17, 2), Swedish (32, 1)
12	f	28	13	1	b2	French (13, 4), Spanish (15, 4)
13	f	32	15		c1	French (12, 2), Spanish (28, 1)
14	m	37	13		b2	French (11, 3), Italian (15, 3)
15	f	46	12		c1	French (3, 4), Italian (11, 4)
16	m	42	15		c1	French (15, 1)
17	f	33	16		c2	French (13, 4)
18	f	38	12		c1	French (11, 4), Amharic (23, 3), Tamil (16, 3), Romanian (23, 1)
19	f	38	13		c1	French (10, 4), Italian (16, 4), Spanish (21, 3), Indonesian (23, 1), Russian (24, 1)
20	f	40	12		c2	French (4, 3), Italian (22, 3), Spanish (29, 4)
21	m	37	13		c1	Turkish (3, 4), French (11, 4)
22	f	40	13		c1	French (13, 4), Italian (16, 3), Spanish (24, 2)
23	f	48	14		c1	Spanish (23, 2), French (23, 1)
24	f	37	11		b2	French (12, 4)

*AOA [E], age of English acquisition; Listening [E], English listening comprehension proficiency, according to the common European framework of reference for languages (ranging from a1 to c2). AOA [foreign languages], Age of acquisition of foreign languages: the first number between brackets refers to the AOA, the second one to the self-estimated proficiency in the range from 1 to 6*.

### Semantic Decision Task

In the context of a block design (i.e., each language direction was presented in a different block), the participants performed four runs of a mixed [German-English (GE) and English-German (EG)] and unmixed [German-German (GG) and English-English (EE)] semantic decision task, consisting in judging whether two consecutive presented disyllabic nouns are semantically congruent (67 word pairs in each language direction; e.g., EE: journey-travel; GG: Fahrstuhl-Aufzug; GE: Flasche-bottle; EG: damage-Schaden) or incongruent (67 word pairs in each language direction; e.g., EE: traffic-poison; GG: Ampel-Rücken; GE: König-surprise; EG: flower-Hafen) by pressing the corresponding response keys (Elmer et al., 2010).

### Stimulus Material and Procedure

The linguistic stimuli were used in a previous work of our group (Elmer et al., 2010), spoken by a bilingual German-Canadian female speaker, registered as 16 bit stereo files, and matched for intensity by using the Adobe Audition software (Adobe Audition 1.5). The stimuli were presented binaurally with a sound pressure level of about 50 dB (SPL, Digital Sound Level Meter 329, Voltcraft) by using HIFI-headphones (Sennheiser, HD 25–1, 70 Ω, Ireland), matched for syllables length (disyllabic nouns), word frequency^3^, and double checked by a professional linguist for plausibility. The auditory stimuli had a mean duration of 800 ms, the inter-stimulus interval between noun-pairs was of 1600 ms, and the inter-trial interval corresponded to 1200 ms after response selection (Elmer et al., 2010). Stimulus presentation and recording of responses were controlled by the Presentation software.^4^

### EEG Data Acquisition and Pre-Processing

Participants seated in a dimmed and acoustically shielded room at about 110 cm distance from a monitor and were instructed to fixate a cross presented at the center of the screen in order to minimize eye movements during EEG recording. Continuous EEG (57 electrodes + 2 eye channels, provided by Easy Cap) was recorded with a sampling rate of 500 Hz and a high pass filter of 0.1 Hz by using an EEG-amplifier (Brainproducts, Munich, Germany). The electrodes (sintered silver/silver-chloride) were located at frontal, temporal, parietal and occipital scalp sites according to the international 10–10 system (Fp1, Fp2, AF3, AF4, F7, F5, F3, F1, Fz, F2, F4, F6, F8, FT7, FC5, FC3, FC1, FCz, FC2, FC4, FC6, FT8, T7, C5, C3, C1, Cz, C2, C4, C6, T8, TP7, CP5, CP3, CP1, CPz, CP2, CP4, CP6, TP8, P7, P5, P3, P1, Pz, P2, P4, P6, P8, PO7, PO3, POz, PO4, PO8, O1, Oz, O2). The online reference electrode was placed over the left mastoid (TP9), and electrode impedance was reduced to <10 kΩ by using electrogel conductant. For all pre-processing steps, we used the Brain Vision Analyser Software Package (Version 2.01, Brainproducts, Munich, Germany). Data were re-referenced offline to an average reference, filtered from 0.1 to 30 Hz, and artifacts were corrected by using an independent component analysis (ICA; Jung et al., 2000) in association with a semi-automatic raw data inspection. After data pre-processing and baseline correction relative to the −100 to 0 ms prestimulus time period, the brain responses to the first word, as well as the ISI period (central period between the two single nouns), were segmented into single sweeps of 800 ms, and data were subjected to intracranial functional connectivity analyses by using the eLORETA toolbox. Functional connectivity in response to the second word was not evaluated because more likely reflecting semantic matching processes than sensory-to-articulation coupling mechanisms.

### Intracranial Functional Connectivity Analyses

Functional connectivity was evaluated in the same way as in a previous work of our group (Elmer et al., 2015) by estimating intracranial lagged phase-synchronization and by using the eLORETA Software Package.^5^ Lagged phase synchronization is a measure for the similarity (a corrected phase synchrony value) between signals in the frequency domain based on normalized (unit module) Fourier transforms; thus it is related to nonlinear functional connectivity. This lagged connectivity measure is accurately corrected as it represents the connectivity between two signals after the instantaneous zero-lag contribution has been excluded (Nolte et al., 2004; Stam and van Straaten, 2012). Such a correction is preferable when using scalp EEG signals or estimated intracranial signals (EEG tomography), because zero-lag connectivity in a given frequency band is often due to non-physiological effects or intrinsic physics artifacts. In particular, volume conduction and low spatial resolution that usually affect other connectivity indices. Thus, this measure is thought to contain only true physiological connectivity information.

In the current implementation of eLORETA, computations were made within a realistic head model (Fuchs et al., 2002), using the MNI 152 template (Mazziotta et al., 2001), with the three-dimensional solution space restricted to cortical gray matter, as determined by the probabilistic Talairach atlas (Lancaster et al., 2000). The intracranial volume is partitioned in 6239 voxels at 5 mm spatial resolution. eLORETA images represent the electric activity at each voxel in neuroanatomic Montreal Neurological Institute (MNI) space as the exact magnitude of the estimated current density. Anatomical labels as Brodmann areas (BA) are reported using MNI space, with correction to Talairach space (Brett et al., 2002).

**Figure 1:**
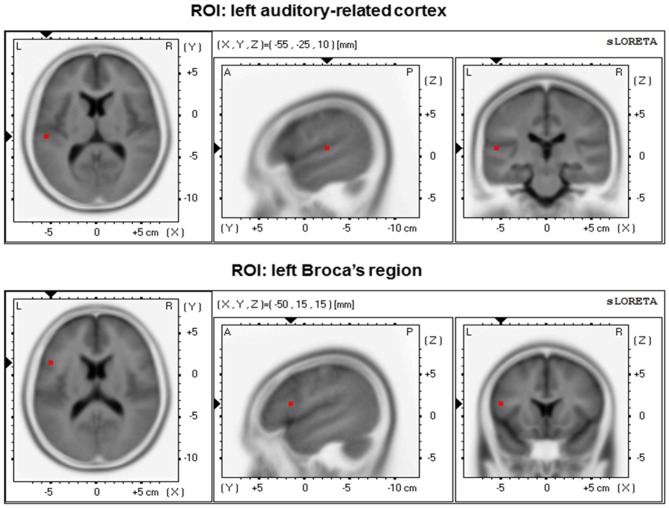
**Regions of interest (ROIs) position within the 3D MNI space.** Left: transversal view; middle: sagittal view; right: coronal view.

For intracranial functional connectivity analyses, we selected two a-priori defined regions of interest (ROIs) in the left hemisphere (Figure 1). These two ROIs consisted of BA 41/42 (ROI 1, left ARC) and left BA 44/45 (ROI 2, Broca’s region). Intracranial connectivity between the two left-sided ROIs was evaluated based on a specific a-priori hypothesis. In addition, the same two ROIs in the right hemisphere were used as control regions. For functional connectivity analyses between the two ROIs, a method using a single voxel at the centroid of the BAs was chosen (Elmer et al., 2015). Details on eLORETA connectivity algorithms can be found in previous reports by Pascual-Marqui et al. (2011). For each group, eLORETA functional connectivity was only computed in the theta frequency band (θ; ~4–7 Hz). In fact, previous work has repeatedly shown that theta oscillations constitute salient markers of information integration (Ward, 2003) and neuronal communication between distinct brain regions over long-range circuits (Ward, 2003; Polanía et al., 2012; Elmer et al., 2015).

### Statistical Analyses

Prior to statistical analyses, all data (autobiographical-, behavioral-, and EEG data) were tested for normal distribution by using the Kolmogorov-Smirnov procedure (*p* > 0.25). Normal The data were analyzed by using analyses of variance (ANOVA) as well as parametric correlations (according to Pearson’s *r*). Since the cumulative number of training hours as well as functional connectivity is dependent on age (Satterthwaite et al., 2013), the relationship between these two variables was assessed by using partial correlations (i.e., corrected for the influence of age). English listening comprehension was evaluated by means of a Mann-Whitney-U test.

## Results

### Autobiographical and Behavioral Data

The two groups did not differ in age (*t*_(22)_ = −0.452, *p* = 0.656, two-tailed), in age of L2 acquisition (*t*_(22)_ = −0.971, *p* = 0.342, two-tailed), nor in L2 listening comprehension (i.e., proficiency level from a1 to c2 was coded numerically from 1 to 6; Mann-Whitney-*U* = 62, *p* = 0.488, two-tailed).

**Figure 2:**
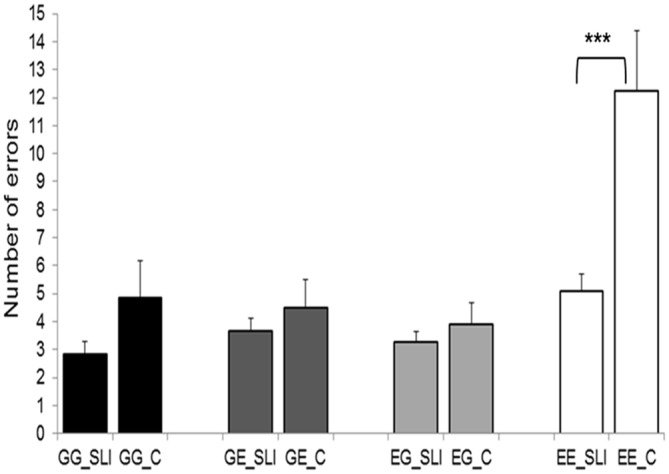
**Behavioral results of the semantic decision task.** SLI, simultaneous language interpreters; C, multilingual control participants; GG, German-German; GE, German-English; EG, English-German; EE, English-English. Error bars depict standard error. ***p < 0.001.

Error scores during the semantic decision task were evaluated by a 2 × 4 ANOVA (2 groups; 4 language directions). This procedure revealed significant effects of “language direction” (*F*_(1,22)_ = 28.142, *p* = 0.000025) and “group” (*F*_(1,22)_ = 7.935, *p* = 0.010) as well as a significant “language direction” × “group” interaction effect (*F*_(1,22)_ = 8.439, *p* = 0.008). *Post hoc* *t*-tests for independent samples only reached significance in the language direction English-English (EE: *t*_(22)_ = −3.723, *p* = 0.001; EG: *t*_(22)_ = −0.921, *p* = 0.367; GE: *t*_(22)_ = −0.875, *p* = 0.391; GG: *t*_(22)_ = 0.054, *p* = 0.12; two–tailed). Taken together, the most errors were committed in the EE condition, whereas the main effect of “group” as well as the “group” × “language direction” interaction effect originated from a better performance of SIs during the EE condition (Figure 2).

### Intracranial Functional Connectivity

**Figure 3:**
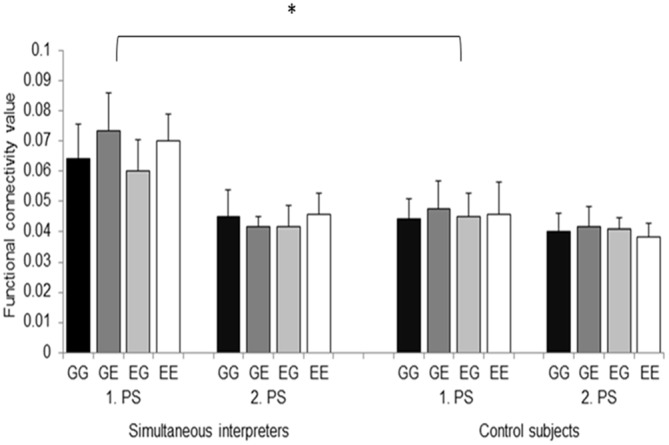
**Functional connectivity values between the left ARC and Broca’s region are depicted separately for the 2 groups, the 2 processing stages (i.e., 1. PS and 2. PS), and the 4 language directions.** GG, German-German; GE, German-English; EG, English-German; EE, English-English. Error bars indicate standard error. *p < 0.05.

Functional connectivity data were subjected to an omnibus 2 × 2 × 2 × 4 ANOVA [2 groups, 2 hemispheres, 2 processing stages (i.e., first word and ISI), and 4 language directions]. Results revealed a main effect of “processing stage” (*F*_(1,22)_ = 26.266, *p* = 0.001) as well as a significant “group” × “hemisphere” × “processing stage” interaction (*F*_(1,22)_ = 5.511, *p* = 0.028). Additional separate 2 × 2 × 4 analyses for each hemispheres (2 groups, 2 processing stages, and 4 language directions] revealed a main effect of “group” (*F*_(1,22)_ = 4.908, *p* = 0.037), of “processing stage” (*F*_(1,22)_ = 15.907, *p* = 0.001), as well as a significant significant “group” × “processing stage” interaction effect (*F*_(1,22)_ = 6.959, *p* = 0.015) in the left hemisphere. Figure 3 indicates that the main effect of “group” as well as the significant “group” × “processing stage” interaction effect originate from an increased left-sided functional connectivity in the SIs group while processing the first word (i.e., processing stage 1), irrespective of language direction (i.e., mixed or unmixed conditions). The main effect of “processing stage” was related to an increased θ phase alignment in the left dorsal pathway in response to the first word, irrespective of group affiliation. By contrast, right-hemispheric analysis revealed a significant main effect of “processing stage” that originated from increased connectivity during processing stage 1 (*F*_(1,22)_ = 14.254, *p* = 0.001), irrespective of group.

### Relationships Between Intracranial Connectivity and Biographical Data

**Figure 4:**
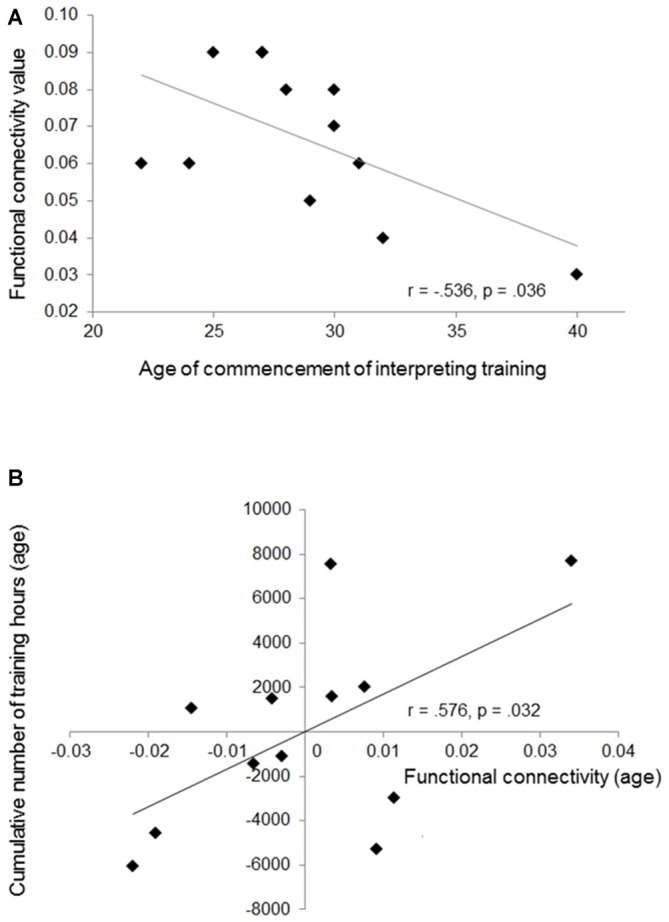
**This figure shows the negative correlation between mean functional connectivity and age of training commencement (A), and the positive relationship between cumulative number of training hours and mean connectivity (partial correlation) (B).** The partial correlation is depicted by plotting residual values of training (age) against connectivity (age).

Based on the results, we additionally investigated putative relationships between mean functional connectivity during the first processing stage (mean connectivity values across all language directions) and age of commencement of interpreting training. Furthermore, in order to address training-related changes in functional connectivity, we performed partial correlations (corrected for the influence of age) between left-hemispheric connectivity and the cumulative number of training hours across lifespan. Results demonstrate a negative relationship between age of commencement and mean functional connectivity (Person’s *r*, *r* = −0.536, *p* = 0.036, one-tailed). In addition, we revealed a significant positive correlation between mean connectivity and the cumulative number of training hours across lifespan (partial correlation, *r* = 0.576, *p* = 0.032). These results indicate that the increased functional connectivity we observed in professional SIs results from an intertwining of sensitive period and training intensity (Figure 4). However, since from Figure 4 it becomes visible that one SI started quite late with interpreting, we re-performed all correlative analyses by excluding this specific individual. Results still revealed a significant relationship between the cumulative number of training hours and mean left-sided connectivity (*r* = 0.643, *p* = 0.023), whereas the correlation between age of commencement and mean connectivity failed to reach significance (*r* = −0.242, *p* = 0.237).

## Discussion

The main assumption of the present work was that the strong demands placed on sensory-to-articulation mapping mechanisms in SIs will have a distinctive influence on the temporal alignment of neural oscillations between the left ARC and Broca’s region. Therefore, here we evaluated θ phase synchronization between these two main hubs of the dorsal stream in both hemispheres while participants performed a mixed and unmixed auditory semantic decision task. Usually, in similar linguistic conditions brain activity spreads along the ventral processing stream by initiating a cascade of neuronal processes mirroring lexical-semantic representations (Visser and Lambon Ralph, 2011; Marconi et al., 2013). However, due to extensive and specific practice of SIs, here we predicted that this specific subgroup of participants will more strongly pre-activate the dorsal stream that is necessarily involved when it comes to anticipate the articulatory code of the corresponding translation (Price et al., 1999; Quaresima et al., 2001). Exactly this perspective is supported by our results. In fact, EEG data revealed a significant “group” × “hemisphere” × “processing stage” interaction effect (Figure 3), suggesting an earlier and more pronounced recruitment of the left dorsal stream in SIs. Most notably, functional connectivity strengths significantly correlated with the age of training commencement and the cumulative number of training hours, indicating training-related changes as well as the influence of sensitive periods. Finally, the same analysis also yielded a main effect of “processing stages” irrespective of group affiliation, a result that reinforces the general mutual interdependence of the speech perception and production systems (Liberman and Mattingly, 1985).

Certainly, simultaneous translation constitutes a linguistic task that places demands on the ventral pathway in order to access lexical-semantic knowledge in the target language. However, this is even true for multilingual participants, especially when they do not differ in age of L2 acquisition and are characterized by a comparable L2 proficiency level (Perani and Abutalebi, 2005; Elmer et al., 2010). By contrast, the continuous conveyance of an input into a target language with a minimal time delay (while at the same time adapting the linguistic structure of the output sentence) can hardly be achieved by multilingual speaker who are not specifically trained in such a complex linguistic task. Consequently, it appears natural to assume that simultaneous translation places more distinctive functional demands on the dorsal processing stream, a circuit that is not only involved in sound-to-articulation mapping (Price et al., 1999; Rinne et al., 2000; Hickok and Poeppel, 2007) but also in the coordination of syntactical aspects of speech in a time-dependent manner (Bornkessel-Schlesewsky and Schlesewsky, 2013). Since results demonstrate an overall increased functional connectivity in SIs, irrespective of language direction, here we propose that the functional coupling between the left ARC and Broca’s region more likely reflects a domain-general process that originates from the time constraints placed on sound-to-articulation synchronization mechanisms rather than linguistic functions *per se*. In particular, we propose that professional interpreting training leads to an automatic co-activation of these two brain regions which are involved in a variety of linguistic, articulatory, and cognitive processes.

It results obvious that the extreme processing demands placed on simultaneous language translation cannot simply be considered as a magnification of the processing requirements engaged in multilingual speech processing. Otherwise, this perspective enables to set important premises for better understanding the neuronal circuits underlying speech processing in association with expertise. In this context, important steps forward have been made regarding the cortical representation of multiple languages (for an overview consider Sebastian et al., 2011) as well as the associated cognitive control mechanisms that are necessarily engaged for avoiding interferences between the different languages (Rodriguez-Fornells et al., 2006; Abutalebi and Green, 2007; Bialystok and Poarch, 2014; Costa and Sebastián-Gallés, 2014).

Most relevant for the the present work are especially studies providing evidence for a selective influence of multilingual speech processing and language expertise on the functional-anatomical architecture of the dorsal part of the ARC and Broca’s region. This perspective is driven by the reasoning that the ramification of multilingual speech processing on the functional-anatomical malleability of these two specific brain regions constitutes the bearing skeleton beyond the increased phase alignment we observed in SIs. In this context, there are at least some studies in earnest showing such an influence. For example, Simmonds et al. (2011) performed fMRI measurements while bilingual participants articulated concatenated sentences in L1 and L2. Results revealed an increased blood-oxygen-level dependent (BOLD) response in the ARC while articulating in L2 compared to L1, possibly reflecting increased demands placed on the integration of predictive feedforward and post-articulatory feedback processes (Simmonds et al., 2011). In addition, by evaluating brain structures in phoneticians and non-phoneticians, Golestani et al. (2011) observed a relationship between expertise and the morphology of the ARC. In the same work, the researchers also reported a positive correlation between the volume of left Broca pars opercularis and the number of years of phonetic transcription training. Finally, at least other two studies (Golestani et al., 2007; Wong et al., 2008) reported a positive correlation between the ability to perceive foreign speech sounds and ARC volume, and a further one found evidence for plasticity effects in the ARC of bilingual participants (Ressel et al., 2012). Certainly, the results of these previous studies cannot be directly compared to interpreting training. However, they provide at least evidence for brain changes in the two main hubs of the dorsal pathway in association with expertise in different domains.

There is evidence showing that language translation is strongly dependent on the recruitment of the left dorsal pathway, in both SIs (Rinne et al., 2000; for a comprehensive review on SIs consider Elmer, 2012) and multilingual participants (Price et al., 1999; Hervais-Adelman et al., 2015). Previous work (Elmer et al., 2014) more specifically focusing on plasticity effects in SIs (compared to multilingual control participants) reported (among other areas) an altered gray matter architecture in Broca’s region and in the left supramarginal gyrus, the latter being part of ARC (Caspers et al., 2006; for comparable results in bilinguals consider Golestani and Pallier, 2007). Most interestingly, the volume of Broca’s area correlated with the cumulative number of training hours across lifespan, indicating training-related changes. In a further DTI study performed with the same population (Elmer et al., 2011), the same authors found white matter differences between SIs and multilingual control participants in the left supramarginal gyrus (for comparable results in bilinguals consider Mechelli et al., 2004) as well as in the extreme capsule, a fiber tract that connects Broca’s area with the left anterior insula, a brain structure that has been proposed to support (among other functions) articulation of speech and phonation (Eickhoff et al., 2009; Ackermann and Riecker, 2010). Finally, a recent EEG study (Elmer et al., 2014) brought to light differential event-related brain responses to morphed speech stimuli in SIs, compared to control participants, during early stages of auditory processing. An alternative plausible interpretation of the results is that due to intensive training SIs require fewer demands to perform the task and that the automatic recruitment of the dorsal stream reflects higher dexterity. This perspective is partially supported by the behavioral data showing that SIs committed fewer errors during the task, especially in the EE condition. Furthermore, we cannot exclude that the increased functional connectivity we observed in SIs may reflect a top-down control of audition (Geranmayeh et al., 2014) or even higher prediction processes.

In summary, based on our results, we propose that in SIs “hearing” and “speaking” are tightly coupled in time, in such a manner that this distinctive and training-related electrophysiological signature can saliently be detected by evaluating functional connectivity in the left dorsal pathway. This is an important step toward a holistic and integrative understanding of the speech perception and production systems in the context of multilingual speech processing. Certainly, the reciprocal influence of the ventral and dorsal pathway on the two main hubs described, as well as the contribution of working memory functions, have to be described in more detail. The same is true for the influence of auto-biographical and genetic features and its relationships to functional and structural plasticity.

## Author Contributions

SE collected the data, performed the statistical analyses, and wrote the manuscript. JK was involved in the connectivity analyses.

## Conflict of Interest Statement

The authors declare that the research was conducted in the absence of any commercial or financial relationships that could be construed as a potential conflict of interest.

## Footnotes

^1^http://www.lancaster.ac.uk/researchenterprise/dialang/dialang_technical

^2^http://www.esseraudio.com/de/home-audiometer-hoertest.html

^3^http://www.corpora.uni-leipzig.de/

^4^http://www.neurobs.com

^5^http://www.uzh.ch/keyinst/loreta.htm
